# Blood Pressure Gradients and Cardiovascular Risk Factors in Urban and Rural Populations in Abia State South Eastern Nigeria Using the WHO STEPwise Approach

**DOI:** 10.1371/journal.pone.0073403

**Published:** 2013-09-05

**Authors:** Ikechi Gareth Okpechi, Innocent Ijezie Chukwuonye, Nicki Tiffin, Okechukwu Ojoemelam Madukwe, Ugochukwu Uchenna Onyeonoro, Theophilus Ifeanyichukwu Umeizudike, Okechukwu Samuel Ogah

**Affiliations:** 1 Division of Nephrology and Hypertension, Groote Schuur Hospital and University of Cape Town, South Africa; 2 Division of Nephrology, Department of Medicine, Federal Medical Centre, Umuahia, Abia State, Nigeria; 3 South African National Bioinformatics Institute/MRC Unit for Bioinformatics Capacity Development, University of the Western Cape, Cape Town, South Africa; 4 Ministry of Health, Nnamdi Azikiwe Secretariat, Umuahia, Abia State, Nigeria; 5 Department of Community Medicine, Federal Medical Centre, Umuahia, Abia State, Nigeria; 6 Nephrology Unit, Department of Medicine, Lagos State University Teaching Hospital, Ikeja Lagos, Nigeria; 7 Division of Cardiovascular Medicine, Department of Medicine, University College Hospital, Ibadan, Oyo State, Nigeria; John Hopkins bloomerg school of public health, United States of America

## Abstract

**Background:**

Developing countries of sub-Saharan Africa (SSA) face a double burden of non-communicable diseases (NCDs) and communicable diseases. As high blood pressure (BP) is a common global cardiovascular (CV) disorder associated with high morbidity and mortality, the relationship between gradients of BP and other CV risk factors was assessed in Abia State, Nigeria.

**Methods:**

Using the WHO STEPwise approach to surveillance of chronic disease risk factors, we conducted a population-based cross-sectional survey in Abia state, Nigeria from August 2011 to March 2012. Data collected at various steps included: demographic and behavioral risk factors (Step 1); BP and anthropometric measurements (Step 2), and fasting blood cholesterol and glucose (Step 3).

**Results:**

Of the 2983 subjects with complete data for analysis, 52.1% were females and 53.2% were rural dwellers. Overall, the distribution of selected CV disease risk factors was diabetes (3.6%), hypertension (31.4%), cigarette smoking (13.3%), use of smokeless tobacco (4.8%), physical inactivity (64.2%) and being overweight or obese (33.7%). Presence of hypertension, excessive intake of alcohol, smoking (cigarette and smokeless tobacco) and physical inactivity occurred more frequently in males than in females (p<0.05); while low income, lack of any formal education and use of smokeless tobacco were seen more frequently in rural dwellers than in those living in urban areas (p<0.05). The frequency of selected CV risk factors increased as BP was graded from optimal, normal to hypertension; and high BP correlated with age, gender, smokeless tobacco, overweight or obesity, annual income and level of education.

**Conclusion:**

Given the high prevalence of hypertension in this part of Nigeria, there is an urgent need to focus on the reduction of preventable CV risk factors we have observed to be associated with hypertension, in order to effectively reduce the burden of NCDs in Africa.

## Introduction

Developing countries of the world, especially in sub-Sahara Africa (SSA) face a double burden of communicable diseases (e.g. HIV/AIDS, malaria and tuberculosis) and chronic non-communicable diseases [NCDs] (e.g. diabetes, hypertension, cancer and chronic renal failure) [1]. As much attention continues to be given to the diagnosis, treatment and eradication of communicable diseases, the prevalence of many NCDs in these countries have continued to increase unfettered. Rapid urbanization, often involving lifestyle changes (high salt and fat diet, cigarette smoking, increased alcohol intake and lack of exercise) may partly explain the epidemiological transition in SSA away from infectious diseases, towards NCDs as leading causes of death [2], [3]. It is estimated that NCDs may account for up to 60–64% of all deaths in low- and middle- income countries (LMIC); and that by the year 2015 over 70% of all deaths worldwide will be attributable to a non-communicable cause [1], [4]–[6]. In South Africa, one study has reported an increasing burden of NCDs in rural communities as well as a disproportionate burden of NCDs in poor people who live in urban settings [7].

Cardiovascular diseases (CVD) including hypertension, coronary artery disease, cerebrovascular disease, cardiomyopathies, rheumatic heart diseases, etc., are a major component of NCDs and are now emerging or rapidly increasing in many developing countries [8]. Cardiovascular diseases are sustained by risk factors like use of tobacco and alcohol, physical inactivity and unhealthy choices of diet. Of all the CVDs, hypertension is globally the most prevalent as it affects up to 1 billion people, contributing to 7.1 million deaths per year and 92 million disability-adjusted life years [9]. The prevalence of hypertension reported from many SSA countries has been shown to vary extensively between and within studies. Using a BP≥140/90 mmHg as cut-off, the prevalence of hypertension is reported to be between 7.5% in Sudan [10] and 42.2% in Nigeria [11].

The relationship between BP and CV risk is known to be continuous such that CV risk doubles with every 20 mmHg elevation in systolic BP (SBP) or 10 mmHg elevation in diastolic BP (DBP) [12]. There is still paucity of data in SSA assessing the relationship between gradients of hypertension (as defined by various guidelines) and the frequency of selected CV risk factors determined from large population studies. Hence, the purpose of this study is to examine this relationship in rural and urban populations in Abia state, South East of Nigeria using the WHO STEPwise approach [13].

## Materials and Methods

### Ethics Statement

This study was approved by the Abia State Ministry of Health Ethics Review Committee and all the participants provided informed consent in writing, in accordance with the Declaration of Helsinki.

### Study Population

**Figure 1:**
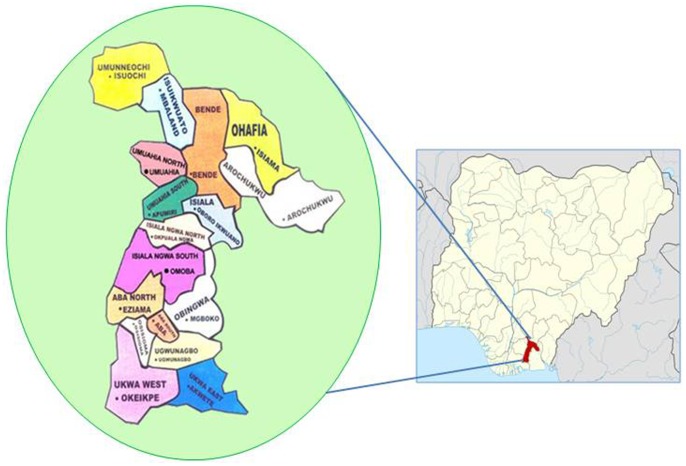
Geographical location of Abia State in Nigeria showing the distribution of local government areas within the state.

Abia state is in the South Eastern part of Nigeria with an estimated population of 3,152,691 people (approximately 2% of the national population) (see Figure 1). It is predominantly inhabited by Igbo people of Nigeria. This study was of cross-sectional design using the WHO STEPwise approach to surveillance in adult men and women aged 18 years and older in Abia state.

We adopted the WHO STEPS guideline (http://www.who.int/chp/steps/resources/sampling/en/) to calculate the appropriate and minimum sample size and the STEPS method to select a representative sample for the state. The level of confidence and the corresponding margin of error (MOE) used for the sample size calculation for the survey were 95% and 0.05 respectively. As there was no previous data on baseline levels of the indicators for the state, an estimated prevalence of 50% was used in order to ensure the most conservative sample size (n_1_). Using the values obtained and the population estimate for each 10-year age group by sex cluster for the population of the state (based on the 2006 population census), the estimated sample size was computed for each age and sex strata (n_2_). The total sample size was then adjusted for design effect (n_3_) and for expected non-response rate (n_4_ or final sample size). The design effect of 1 was chosen (for random sampling) while the expected response rate was 80%.

**Step 1:** n_1_ = Z^2^ p (1-p)/e^2^

Where:

n = sample size

z = level of confidence (1.96 or 95%)

p = baseline level of indicators (0.5 or 50%)

e = margin of error

**Step 2:** n_2_ = n_1/_(1+ [n_1/_population])

**Step 3:** n_3_ = n_2_ * design effect

**Step 4:** n_4_ = n3/response rate.

Hence, the minimum calculated sample size for this study, making allowance for design effect, age-sex estimates as well as non-response rate was 2,880. However, of 2,999 subjects that were interviewed, 2,983 subjects with complete demographic and clinical data were included for analysis in this study. A multistage stratified cluster sampling technique was used to select the study participants over a seven month period (August 2011 to March 2012). Abia state is divided into three senatorial zones: Abia North, Abia Central and Abia South. One rural and one urban local government area (LGA) was randomly selected from each senatorial zone. They were Ohafia and Isuikwuato/Bende for Abia North, Umuahia North and Ikwuano for Abia Central and Aba South and Ukwa East for Abia South Senatorial Zones (Figure 1). In each LGA, four Enumeration Areas (EAs) were randomly selected from the listing of all the EAs. Households in these EAs were further listed and eligible participants were selected. The selection was such that not more than two eligible participants of either sex were selected from each household. Using the EA map and starting from a prominent landmark in the community (e.g. church, school, police station etc), trained interviewers proceeded from household to household; interviewing eligible listed respondents until a minimum of 120 respondents were interviewed in the community.

### Study Questionnaire

The WHO-STEPwise approach surveillance questionnaire was used for data collection, and was administered by a team of trained health workers comprising of six interviewers and supervisors. The supervisors were public health physicians, a cardiologist and a nephrologist. Information collected included socio-demographic parameters such as gender, age, use of alcohol and tobacco and dietary information on consumption of fruits and vegetables. Other data recorded includes personal history and family history of chronic NCDs such as hypertension, diabetes, cancer, asthma and awareness of common NCDs and physical activity.

### Anthropometry and Blood Pressure Measurement

Weight and height were measured and used to assess the body mass index (BMI). Body mass index was categorized using standard cut-off values. Blood pressure measurements were performed with an Omron Digital Blood Pressure machine (Omron M2 automatic BP monitor - Tokyo Japan) which was battery powered. Blood pressure was measured with subjects sitting and after resting for 5 minutes and with their legs uncrossed. Three BP reading at intervals were taken; the average of the second and third readings was used for analysis. Systemic hypertension was defined as SBP≥140 mmHg and or DBP≥90 mmHg or normal SBP and DBP in a subject using antihypertensive treatment [14]. Gradients of BP into “optimal”, “normal”, “pre-hypertension”, “stage 1”, “stage 2” and “stage 3” hypertension was defined according to guideline recommendations [14]–[16].

### Biochemical Analysis

Although blood was drawn in the fasting state for assessment of glucose and fasting lipogram, only fasting glucose was evaluated in all the subjects. Fasting lipogram could only be performed in 168 subjects, i.e. 5.6% of the entire study population and was therefore not used in analysis of our data.

### Data Analysis

Data was analyzed using SPSS statistical software (SPSS Inc, Chicago Illinois, USA). Data was tested for normality using the Kolmogorov-Smirnov Test. All the continuous variables were found to be normally distributed. Continuous variables were reported as mean ± standard error of mean (SEM) while categorical variables were reported as percentages. Pearson correlation was used to test association of hypertension (presence or absence) with various demographic variables including age, gender, place of domicile, smoking, alcohol use, physical inactivity etc. Significant variables were entered into a multiple regression to determine the independent predictors of hypertension and the 95% confidence intervals were reported when appropriate. A p-value of <0.05 was taken as significant.

## Results

### Features of the Study Participants Enrolled into the Study

Participants in this study were enrolled from all the enlisted 24 EAs of the six LGAs chosen for this study. Although a total of 2,999 subjects were enrolled, complete data was available only in 2,983 of the subjects who were included in this analysis. There was a slight preponderance of female subjects in this study (52.1%) and a slightly higher sampling of subjects in rural areas (53.2%). The mean age at time of interview was 41.7±0.3 years (41.5±0.5 years for males and 41.8±0.5 years for females) with 47.8% of all those sampled falling within the age group of 20–39 years old; similar to the proportion of this age group in the Abia state adult population. There were 319 people (10.7%) without any formal education and 2011 people (67.4%) were actively employed. The level of illiteracy was higher in rural dwellers and in females (Table 1).

**Table 1:** Demographic features of participants enrolled in the Abia State NCD STEPS survey.

Factor	Overall (n = 2983)	Urban (n = 1396)	Rural (n = 1587)	Male (n = 1430)	Female (n = 1553)
Male	1430 (47.9)	699 (50.1)	731 (46.1)	1430 (100)	–
Female	1553 (52.1)	697 (49.9)	856 (53.9)	–	1553 (100)
Age (years)	41.7±0.3	39.3±0.5	43.9±0.5	41.5±0.5	41.8±0.5
<20 years	168 (5.6)	84 (6.0)	84 (5.3)	82 (5.7)	86 (5.5)
20–29 years	864 (29.0)	449 (32.2)	415 (26.2)	432 (30.2)	432 (27.8)
30–39 years	561 (18.8)	294 (21.1)	267 (16.8)	255 (17.8)	306 (19.7)
40–49 years	389 (13.0)	199 (14.3)	190 (12.0)	197 (13.8)	192 (12.4)
50–59 years	380 (12.9)	139 (10.0)	241 (15.2)	173 (12.1)	206 (13.3)
60–69 years	282 (9.5)	108 (7.7)	174 (11.0)	131 (9.2)	151 (9.7)
≥70 years	312 (10.5)	111 (8.0)	201 (12.7)	153 (10.7)	159 (10.2)
Highest level of education					
None	319 (10.7)	108 (7.7)	211 (13.3)	72 (5.0)	247 (15.9)
Primary	731 (24.5)	261 (18.7)	470 (29.6)	374 (26.2)	357 (23.0)
Secondary	1395 (46.8)	668 (47.9)	727 (45.8)	695 (48.6)	700 (45.1)
Tertiary	538 (18.0)	359 (25.7)	179 (11.3)	289 (20.2)	249 (16.0)
Occupation					
Employed	2011 (67.4)	916 (65.6)	1095 (69.0)	1011 (70.7)	1000 (64.4)
Studying/Apprentice	403 (13.5)	217 (15.5)	186 (11.7)	212 (14.8)	191 (12.3)
Retired	139 (4.7)	57 (4.1)	82 (5.2)	70 (4.9)	69 (4.4)
Unemployed	430 (14.4)	206 (14.8)	224 (14.1)	137 (9.6)	293 (18.9)

Data is presented as mean ± standard error of mean (SEM) or as number of subjects (percentage).

### The Extent of Selected Risk Factors for Non-communicable Diseases in Abia State, Nigeria

Diabetes mellitus was present in 3.6% of all those assessed and this was similar in distribution for males and females and those in urban or rural areas (P>0.05). Overall, high intake of alcohol, inadequate consumption of fruits and vegetables and low physical activity were commonly observed (46.9, 70.4 and 64.2% respectively). Alcohol consumption (including daily intake and excessive use of alcohol) was significantly higher in males than in females (66.4% vs 28.9%; P<0.05). There was a significantly higher proportion of females that were overweight or obese compared to males (37.0% vs 30.1%; P<0.05) and a higher prevalence of overweight and obesity in urban residents compared to rural inhabitants (37.5% vs 30.4%; P<0.05) (Table 2).

**Table 2:** Distribution of selected NCDs and their risk factors in participants enrolled in the Abia State NCD STEPS survey.

Factor	Overall (n = 2983)	Urban (n = 1396)	Rural (n = 1587)	Male (n = 1430)	Female (n = 1553)
Diabetes§	107 (3.6)	61 (4.4)	46 (3.0)	51 (3.6)	56 (3.6)
Hypertension	936 (31.4)	429 (30.7)	507 (32.0)	499 (34.9)*	436 (28.1)
Alcohol	1399 (46.9)	669 (47.9)	730 (46.0)	950 (66.4)*	449 (28.9)
Smoking (Ex- and current)	398 (13.3)	184 (13.2)	214 (13.5)	383 (26.8)*	15 (1.0)
Use of smokeless tobacco	142 (4.8)	42 (3.0)#	100 (6.3)	103 (7.2)*	39 (2.5)
Inadequate intake of fruits and vegetables	2100 (70.4)	975 (69.8)	1125 (70.9)	970 (67.8)*	1126 (72.5)
Physical Inactivity	1914 (64.2)	911 (65.3)	1003 (63.2)	995 (69.6)*	918 (59.1)
Obese or overweight	1006 (33.7)	523 (37.5)#	483 (30.4)	431 (30.1)*	575 (37.0)
Low income (≤100,000/yr)	1665 (55.8)	706 (50.6)#	959 (60.4)	654 (45.7)*	1001 (64.5)
No formal education	282 (9.5)	90 (6.5)#	192 (12.1)	65 (4.6)*	217 (14.0)

§ Represents subjects with self reported diabetes, those with fasting blood glucose ≥7.0 mmol/L or a random blood sugar ≥11.1 mmol/L.

* P<0.05 for males vs females.

# P<0.05 for urban vs rural dwellers.

Data is presented as number of subjects (percentage).

Hypertension was present in 31.4% of the overall population. Although the frequency of hypertension was significantly higher in males than in females (34.9% vs 28.1%; P<0.05), there was no significant difference in the frequency of hypertension in urban and rural areas (Table 2). In both the rural and urban areas, mean systolic blood pressure (SBP) was significantly higher in males than in females and there were significantly more males classified as pre-hypertension or stage 1 hypertension than females in both areas (Table 3). The proportion of subjects with stage 2 and stage 3 hypertension was similar to the overall prevalence in urban and rural males and females.

**Table 3:** Blood pressure and blood pressure classification of participants dwelling in urban and rural parts of Abia state.

Blood pressure class	Overall (n = 2983)	Urban (n = 1396)		Rural (n = 1587)	
		Male n = 699 (%)	Female n = 697 (%)	Male n = 731 (%)	Female n = 856 (%)
Mean SBP (mmHg)	134.3±0.4	136.9±0.7#	130.5±0.8	137.0±0.8*	132.9±0.9
Mean DBP (mmHg)	77.7±0.2	78.4±0.5#	76.5±0.5	78.0±0.5	77.9±0.5
Optimal BP	656 (22.0)	81 (11.6)#	221 (31.7)	105 (14.4)*	249 (29.1)
Normal BP	937 (31.4)	236 (33.8)	205 (29.4)	245 (33.5)	251 (29.3)
Pre-Hypertension	743 (24.9)	230 (33.0)#	138 (19.8)	215 (29.4)*	160 (18.7)
Stage 1 Hypertension	655 (22.0)	189 (27.0)#	124 (17.8)	178 (24.4)*	163 (19.0)
Stage 2 Hypertension	271 (9.1)	62 (8.9)	57 (8.2)	68 (9.3)	84 (9.8)
Stage 3 Hypertension	160 (5.4)	34 (4.9)	29 (4.2)	38 (5.2)	59 (6.9)
Hypertension	936 (31.4)	249 (35.6)#	180 (25.8)	250 (34.2)	256 (29.9)

# P<0.05 for urban males vs females.

* P<0.05 for rural males vs females.

Data is presented as mean ± standard error of mean (SEM) or as number of subjects (percentage).

**Figure 2:**
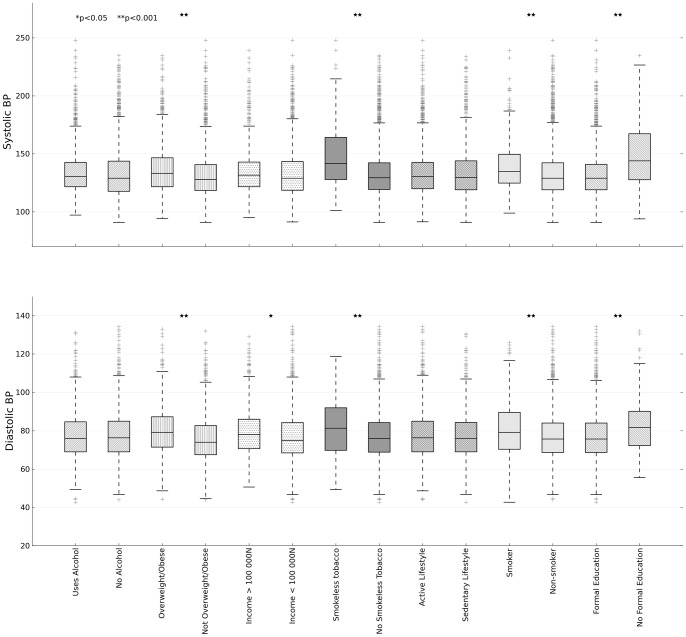
Relationship between selected non-communicable diseases and mean systolic and diastolic blood pressures.

Also, even though hypertension was seen in 58.7% of participants with diabetes, most of the subjects were observed to have systolic hypertension (52.4%) rather than diastolic hypertension (33.3%). This pattern was seen for all the other NCD risk factors assessed in our study: smoking (38.4% vs 23.4%); alcohol (29.4% vs 14.2%); obesity (36.1% vs 18.4%); sedentary lifestyle (31.1% vs 14.3%); inadequate intake of fruits and vegetables (28.6% vs 13.1%), no formal education (55.0% vs 23.4%) and low level annual income (28.1% vs 13.1%) (Table 4). Systolic and diastolic blood pressures were significantly higher in those who used any tobacco product (regular cigarettes [SBP: 139.7±1.6 vs 134.1±0.7; DBP: 80.0±1.0 vs 77.6±0.4] or smokeless tobacco [SBP: 148.1±2.9 vs 133.7±0.6; DBP: 82.1±1.6 vs 77.5±0.4]), those who are overweight or obese [SBP: 137.3±1.1 vs 132.6±0.8; DBP: 80.3±0.6 vs 76.1±0.5] and those with no formal education [SBP: 149.6±2.2 vs 132.6±0.6; DBP: 82.1±1.0 vs 77.3±0.4] (P<0.001) (Figure 2). SBP was not significantly different between the 2 income groups, however those of low income had a slightly lower DBP compared to subjects of the higher income group (P<0.05; Figure 2).

**Table 4:** Scale of selected NCD risk factors in Abia state and their distribution with classes of hypertension.

	Diabetes(n = 107)	Smoking(n = 398)	Smokeless tobacco (n = 142)	Alcohol(n = 1399)	Overweight/Obese (n = 1006)	Physicalinactivity(n = 1914)	Low annual income (n = 1665)	Inadequate fruit and vegetables (n = 2100)		No education (n = 282)
Optimal BP	10 (9.5)	55 (13.8)	11 (7.7)	259 (18.5)	182 (18.1)	410 (21.4)	398 (23.9)		482 (23.0)	36 (12.8)
Normal BP	29 (27.0)	110 (27.6)	35 (24.6)	472 (33.7)	294 (29.2)	610 (31.9)	495 (29.7)		670 (31.9)	72 (25.5)
Hypertension	63 (58.7)	165 (41.5)	78 (54.9)	437 (31.2)	386 (38.4)	605 (31.6)	493 (29.6)		632 (30.1)	159 (56.4)
Systolic hypertension	56 (52.4)	153 (38.4)	75 (52.8)	412 (29.4)	363 (36.1)	596 (31.1)	468 (28.1)		600 (28.6)	155 (55.0)
Diastolic hypertension	36 (33.3)	93 (23.4)	40 (28.1)	199 (14.2)	185 (18.4)	273 (14.3)	223 (13.4)		276 (13.1)	66 (23.4)

Data is presented as number of subjects (percentage).

### Correlates of Hypertension in Abia State, Nigeria

Table 5 shows the correlation between hypertension and selected NCDs in this study. We observed a strong and significant correlation between hypertension and age, gender, use of smokeless tobacco, being overweight or obese and level of education (P<0.0001). Hypertension also correlated with annual income (P = 0.025). Cigarette smoking, alcohol use and physical activity did not correlate with hypertension in this population (p>0.05). However, a multivariate regression analysis for hypertension and NCD risk factors, indicated that age (P<0.0001), gender (P = 0.002) and overweight or obesity (P = 0.001) were the predictors of hypertension (Table 6).

**Table 5:** Correlation analysis for hypertension* and selected NCD risk factors in Abia state Nigeria.

Factor	Pearson’s coefficient	P
Age	0.431	<0.0001
Gender	−0.069	<0.0001
Cigarette Smoking	0.005	0.804
Smokeless tobacco	0.110	<0.0001
Alcohol	0.012	0.533
Overweight and obesity	0.121	<0.0001
Annual Income	−0.045	0.025
Level of education	−0.200	<0.0001
Physical activity	0.002	0.937

* subjects were stratified as having hypertension or not having hypertension. Hypertensives were those with SBP and/or DBP≥140 mmHg and/or ≥90 mmHg respectively or taking antihypertensive medications with lower values.

**Table 6:** Regression analysis for hypertension and NCD risk factors in Abia state Nigeria.

Factor	B (S.E.)	t	P
Age	0.012 (0.001)	18.15	<0.0001
Gender	0.072 (0.023)	−3.08	0.002
Overweight and obesity	0.073 (0.021)	3.42	0.001

B – Unstandardized coefficients; S.E. – standard error.

## Discussion

Hypertension is common in adults and remains a major risk factor for both CV and cerebrovascular morbidity and mortality worldwide [17]. There has for long been much neglect of CV diseases in developing countries, as these diseases were often considered not to be a significant problem in developing countries that have perpetually been under the grip of numerous communicable diseases. Recently, an increased awareness of the double burden of disease (communicable plus non-communicable) in developing countries has gathered pace and has led to increased surveillance for NCDs in many of these countries.

The results of the present study are important as they corroborate reports that risk factors for NCDs are common in SSA and with such high prevalence may significantly contribute to the prevalence of CV diseases in the region. Our results also show that certain socio-demographic and cultural factors like poverty (level of income), level of education and use of smokeless tobacco may play a role in the development or worsening of high BP in our population. Our study therefore strengthens the need for regular and frequent surveillance for NCD risk factors in order to increase population awareness, and for early identification of people at high risk for various NCDs.

Our results have also confirmed those of recent large epidemiological studies in Nigeria that have found a high prevalence of hypertension among predominantly rural (20.8%) [18] and low income earners (42.2%) [11]. Despite the reported high prevalence of hypertension, awareness has often been reported to be low. In this study only 40.7% of those found to be hypertensive were aware of having elevated blood pressure. Ulasi et al have reported only 29.4% awareness prior to screening [11] while Dzudie et al observed that 31.7% from major cities in Cameroon were aware of having elevated blood pressures [19]. A high burden of disease and low awareness of hypertension will significantly impact on the incidence and prevalence of morbidity and mortality from cardiovascular diseases. We have previously reported that an unexpectedly high level of target organ damage was present in newly diagnosed hypertensives in Nigeria (retinopathy in 63.8%, nephropathy in 40.7% and echocardiographic left ventricular hypertrophy in 36.7%) [20]. This obviously implies that many in our population with hypertension are likely to first present with CV or renal complication associated with hypertension at the time of first diagnosis of hypertension.

Data from the WHO in 2004 suggest that the estimated age-standardized mortality from CV disease may be up to three times higher in some SSA countries than in some countries of Europe [21]. As many rural populations in SSA are undergoing an epidemiological transition towards a more urban (or Western) way of life, the prevalence of hypertension as well as other CV risk factors in many of these areas may be on the increase. Previous epidemiological studies from many SSA countries have shown higher prevalence of hypertension in urban compared to rural areas. Result of the International Collaborative Study of Hypertension in Blacks [ICSHIB]) in seven populations of West African origin have shown a lower prevalence of hypertension in more rural West Africa where one in seven adults was found to be hypertensive compared with one in three adults in the United States (using the cut-off of 140/90 mm Hg) [22]. Even within West Africa, the ICSHIB study found that the prevalence of hypertension was higher in urban than in rural Cameroon whether blood pressure cut-off was taken as ≥140/90 mmHg (19.1% vs 15.4%) or ≥160/95 mmHg (8.7% vs 6.1%) [22]. Our study shows a reversal of that trend, with a slightly higher prevalence of hypertension in rural compared to urban parts of Abia state (32.0% vs 30.7%). We believe a large part of this can be explained not only by the differences in income, education or use of smokeless tobacco between rural and urban dwellers but by the similarities in their diet (Table 2). Age might also have played a role for the differences in hypertension prevalence between urban and rural dwellers in our study as rural dwellers were older than urban dwellers and age is a strong determinant of hypertension in this study (Tables 1 and 5). In Africa, rural dwellers are frequently peasants with low income and are often assumed to more likely consume a healthy diet rich in fruits and vegetables. This may not always hold true, as this study has shown a similar proportion of urban and rural dwellers who consume inadequate amounts of fruits and vegetables (69.8% vs 70.9%). This could therefore indicate an increased adoption of the unhealthy lifestyles that predispose to hypertension among rural dwellers. Although a recent study from the same region in Nigeria reported a higher prevalence of hypertension in urban dwellers, there was significant under-sampling from the rural area in that study [23].

Finally, in this study we found an inverse relationship between presence of hypertension with level of education and level of income (Table 5). A large study of over 6000 subjects from India that evaluated sociodemographic, biophysical and biochemical factors according to socioeconomic and educational levels, has reported a high clustering of ≥3 CV risk factors mainly in those with low education, occupation and socioeconomic status [24]. Similarly, in a birth cohort study conducted to investigate the contribution of prenatal and antenatal environmental exposures to later-life hypertensive status, Chen et al. found several factors, including poor education (OR = 1.76, P<0.01), as predictors of later life hypertensive status [25]. Several factors such as adverse early life events, unemployment, stressful environment at work, poor public transport, low social support and cohesion, unhealthy dietary habits and poverty have been associated with greater prevalence of CV risks in populations of low socioeconomic status [26]. Despite having plenty of natural resources such as crude oil, poverty is common in Nigeria and together with an assortment of socio-economic problem; the provision by various tiers of government towards health care for the population is usually inadequate. Individuals are therefore often left with inadequate means of livelihood to give sufficient thought to personal health issues until complications set in: many people are therefore too busy trying to make ends meet and are forced to live in a world where they are completely unaware of the health implications of their lifestyles. Oladapo et al have, in an epidemiological study of 2000 rural dwellers in Ibadan South-West of Nigeria, identified a number of patients and health care provider factors that contribute to chronic health issues [18]. On the part of the patients, these factors included difficulties with transportation to clinics, unwillingness to be absent from work hence missing the day’s wage and many other competing issues; while lack of medications, equipment and personnel at the hospitals were contributory factors from the health care providers. A number of these identified factors hinge on poverty and low levels of education. In this study, SBP was not significantly different between the income groups and although DBP was slightly lower in those who earn a low income, close to a third of those categorized as low earners had hypertension (Figure 2 and Table 4). One study of diabetic patients has shown that people with higher educational attainment benefit to a greater extent from brief self-care interventions, while those with lower educational attainment will require more intensive treatment [27]. Whether this could apply to hypertension awareness, treatment and control in our population is currently unknown. However, our study results show that there is a high prevalence of hypertension among those with no formal education and a strong correlation between presence of hypertension and lack of education (Tables 4 and 5).

Although this study may be representative of Abia State and some South-Eastern parts of Nigeria, the data presented here may not be applicable to the entire country with a very large population and that share a great diversity in socio-economic, socio-cultural and socio-demographic features. This is a limitation of this study. A nation-wide population based study on NCD risk factors is therefore warranted in Nigeria. Another limitation of this study was the inability to assess other factors that contribute to cardiovascular diseases such as lipid profiles in all the participants.

In conclusion, the prevalence of hypertension is high in Abia state Nigeria, especially in rural communities. Although we have reported that in this population hypertension is predominantly age and gender related, awareness needs to be created regarding other lifestyle factors for NCDs (e.g. obesity, use of smokeless tobacco and excessive use of alcohol) that contribute to hypertension prevalence. Further longitudinal studies on the impact of various NCD risk factors on societal health are needed in this part of Nigeria and there is an urgent need to address the growing problem of increasing NCDs in rural parts of Nigeria. Although beyond the scope of this study, we feel the National and State governments need do more to reduce poverty and support the education of people in the community, as these factors often become the drivers for NCDs.

## References

[1] Boutayeb A (2006) The double burden of communicable and non-communicable diseases in developing countries. Trans R Soc Trop Med Hyg. 100: 191–199. 10.1016/j.trstmh.2005.07.021 16274715

[2] Unwin N (2001) Non-communicable disease and priorities for health policy in sub-Saharan Africa. Health Policy Plan 16: 351–352. 11739359 10.1093/heapol/16.4.351

[3] Yach D, Hawkes C, Gould CL, Hofman KJ (2004) The global burden of chronic diseases: overcoming impediments to prevention and control. JAMA 291: 2616–2622. 15173153 10.1001/jama.291.21.2616

[4] Alwan A, Maclean DR, Riley LM, d’Espaignet ET, Mathers CD, et al (2010) Monitoring and surveillance of chronic non-communicable diseases: progress and capacity in high-burden countries. Lancet. 376: 1861–1868. 10.1016/S0140-6736(10)61853-3 21074258

[5] Boutayeb A, Boutayeb S (2005) The burden of non communicable diseases in developing countries. Int J Equity Health. 4: 2. 10.1186/1475-9276-4-2 PMC546417 15651987

[6] Mufunda J, Chatora R, Ndambakuwa Y, Nyarango P, Kosia A, et al (2006) Emerging non-communicable disease epidemic in Africa: preventive measures from the WHO Regional Office for Africa. Ethn Dis 16: 521–526. 17682258

[7] Mayosi BM, Flisher AJ, Lalloo UG, Sitas F, Tollman SM, et al (2009) The burden of non-communicable diseases in South Africa. Lancet. 374: 934–947. 10.1016/S0140-6736(09)61087-4 19709736

[8] Reddy KS (2002) Cardiovascular diseases in the developing countries: dimensions, determinants, dynamics and directions for public health action. Public Health Nutrition 5: 231–237. 12027289 10.1079/phn2001298

[9] Lawes CM, Vander Hoorn S, Rodgers A (2008) Global burden of blood-pressure-related disease, 2001. Lancet 371: 1513–1518. 18456100 10.1016/S0140-6736(08)60655-8

[10] Elbagir M, Ahmed K (1990) Blood pressure in a multiracial urban Sudanese community. J Hum Hypertens 4: 621–624. 2096202

[11] Ulasi II, Ijoma CK, Onwubere BJ, Arodiwe E, Onodugo O, et al (2011) High prevalence and low awareness of hypertension in a market population in Enugu, Nigeria. Int J Hypertens 2011: 869675. 21331378 10.4061/2011/869675 PMC3038598

[12] Lewington S, Clarke R, Qizilbash N, Peto R, Collins R (2002) Prospective Studies Collaboration. Age specific relevance of usual blood pressure to vascular mortality: a meta-analysis of individual data for one million adults in 61 prospective studies. Lancet 360: 1903–1913. 12493255 10.1016/s0140-6736(02)11911-8

[13] World Health Organization. Non-communicable Diseases and Mental Health Cluster. Geneva (2005) WHO STEPS Surveillance Manual: The WHO STEPwise Approach to Chronic Disease Risk Factor Surveillance. Geneva: World Health Organization. p.

[14] Seedat YK, Rayner BL (2011) Southern African Hypertension Society (2011) South African hypertension guideline 2011. S Afr Med J 102: 57–83. 22273141

[15] Chobanian AV, Bakris GL, Black HR, Cushman WC, Green LA, et al (2003) Joint National Committee on Prevention, Detection, Evaluation, and Treatment of High Blood Pressure. National Heart, Lung, and Blood Institute; National High Blood Pressure Education Program Coordinating Committee. Seventh report of the Joint National Committee on Prevention, Detection, Evaluation, and Treatment of High Blood Pressure. Hypertension 42: 1206–1252. 14656957 10.1161/01.HYP.0000107251.49515.c2

[16] Mancia G, Laurent S, Agabiti-Rosei E, Ambrosioni E, Burnier M, et al (2009) European Society of Hypertension. Reappraisal of European guidelines on hypertension management: a European Society of Hypertension Task Force document. J Hypertens 27: 2121–2158. 19838131 10.1097/HJH.0b013e328333146d

[17] Kearney PM, Whelton M, Reynolds K, Muntner P, Whelton PK, et al (2005) Global burden of hypertension: analysis of worldwide data. Lancet 365: 217–223. 15652604 10.1016/S0140-6736(05)17741-1

[18] Oladapo OO, Salako L, Sodiq O, Shoyinka K, Adedapo K, et al (2010) A prevalence of cardiometabolic risk factors among a rural Yoruba south-western Nigerian population: a population-based survey. Cardiovasc J Afr 21: 26–31. 20224842 PMC3721297

[19] Dzudie A, Kengne AP, Muna WF, Ba H, Menanga A, et al (2012) CCS investigator group. Prevalence, awareness, treatment and control of hypertension in a self-selected sub-Saharan African urban population: a cross-sectional study. BMJ Open. 2: e001217. 10.1136/bmjopen-2012-001217 PMC3433777 22923629

[20] Salako BL, Ogah OS, Adebiyi AA, Adedapo KS, Bekibele CO, et al (2007) Unexpectedly high prevalence of target-organ damage in newly diagnosed Nigerians with hypertension. Cardiovasc J Afr 18: 77–83. 17497043

[21] Disease and injury country estimates. Geneva: World Health Organization (2010) Available: http://www.who.int/healthinfo/global_burden_disease/estimates_country/en/Accessed 24 November 2012.

[22] Cooper R, Rotimi C, Ataman S, McGee D, Osotimehin B, et al (1997) The prevalence of hypertension in seven populations of west African origin. Am J Public Health 87: 160–168. 9103091 10.2105/ajph.87.2.160 PMC1380786

[23] Ulasi II, Ijoma CK, Onodugo OD (2010) A community-based study of hypertension and cardio-metabolic syndrome in semi-urban and rural communities in Nigeria. BMC Health Serv Res. 10: 71. 10.1186/1472-6963-10-71 PMC2858142 20302648

[24] Gupta R, Deedwania PC, Sharma K, Gupta A, Guptha S, et al (2012) Association of educational, occupational and socioeconomic status with cardiovascular risk factors in Asian Indians: a cross-sectional study. PLoS One. 7: e44098. 10.1371/journal.pone.0044098 PMC3430674 22952886

[25] Chen X, Zhang ZX, George LK, Wang ZS, Fan ZJ, et al (2012) Birth measurements, family history, and environmental factors associated with later-life hypertensive status. Am J Hypertens 25: 464–471. 22297260 10.1038/ajh.2011.262 PMC3309157

[26] Kaplan GA, Keil JE (1993) Socioeconomic factors and cardiovascular disease: a review of the literature. Circulation 88: 1973–1998. 8403348 10.1161/01.cir.88.4.1973

[27] Sacco WP, Bykowski CA, Mayhew LL, White KE (2012) Educational attainment moderates the effect of a brief diabetes self-care intervention. Diabetes Res Clin Pract 95: 62–67. 21992869 10.1016/j.diabres.2011.08.027

